# Mitochondrial Apoptosis Induced by *Chamaemelum Nobile* Extract in Breast Cancer Cells

**Published:** 2016

**Authors:** Hirsa Mostafapour Kandelous, Misha Salimi, Vahid Khori, Noushin Rastkari, Amir Amanzadeh, Mona Salimi

**Affiliations:** a *Physiology and Pharmacology Department, Pasteur Institute of Iran, Tehran, Iran. *; b *Ischemic Disorders Research Center, Golestan University of Medical Sciences, Gorgan, Iran.*; c *Center for Air Pollution Research (CAPR), Institute for Environmental Research (IER), Tehran University of Medical Sciences, Tehran, Iran. *; d *National Cell Bank of Pasteur Institute of Iran, Tehran, Iran.*

**Keywords:** *Chamaemelum nobile*, Apoptosis, Cancer, Proliferation, Mitochondria

## Abstract

*Chamaemelum nobile* (*Asteraceae*) commonly known as 'Roman chamomile' is a medicinal plant used for numerous diseases in traditional medicine, although its anticancer activity is unknown. The present study was carried out to investigate the anticancer as well as apoptotic activity of ethyl acetate fraction of *C. nobile* on different cancerous cell lines. The cells were treated with varying concentrations (0.001- 0.25 mg/mL) of this fraction for 24, 48 and 72 h. Apoptosis induced in MCF-7 cells following treatment with ethyl acetate fraction was measured using Annexin V/PI, flowcytometry and western blotting analysis. The results showed that *C. nobile* ethyl acetate fraction revealed relatively high antiproliferative activity on MCF-7 cells; however, it caused minimal growth inhibitory response in normal cells. The involvement of apoptosis as a major cause of the fraction-induced cell death was confirmed by annexin-V/PI assay. In addition, ethyl acetate fraction triggered the mitochondrial apoptotic pathway by decreasing the Bcl-2 as well as increasing of Bax protein expressions and subsequently increasing Bax/Bcl-2 ratio. Furthermore, decreased proliferation of MCF-7 cells in the presence of the fraction was associated with G2/M phase cell cycle arrest. These findings confirm that ethyl acetate fraction of *C.nobile* may contain a diversity of phytochemicals which suppress the proliferation of MCF-7 cells by inducing apoptosis.

## Introduction

Cancer is a major cause of death worldwide in which deregulated proliferation of abnormal cells leads to disruption of surrounding tissues (1). Based on the report of The International Agency for Research on Cancer (IARC), the specialized cancer agency of the World Health Organization, about 14.9 million cancer cases were identified around the world in 2013, of these 7.7 million cases were in men and 6.9 million in women and further this number is expected to increase to 24 million by 2035 (2). Among different types of cancer, breast cancer is a common malignant tumor in women. Since there are noticeable failures in clinical therapy including radiation, chemotherapy, immunomodulation and surgery in treatment of cancer, a need of alternative strategies in cancer treatment always exists (3). In this regard, many anticancer agents have been developed and several of them are from natural origin (4). 

Medicinal plants have long been used in treatment of different types of diseases due to less toxicity compared with the modern chemotherapy (5). According to the estimation of the World Health Organization (WHO), almost 65% of the world’s inhabitants trust on traditional medicine for their primary health care (6). This is because medicinal plants contain secondary metabolites which could treat various diseases including cancer with less toxic effects (7).

Chamomile as a well-documented medicinal plant in the world was widely used for different diseases. Its usage dates back to ancient Egyptians to relieve fever and sun stroke. In the sixth century, it was used for treatment of insomnia, back pain, neuralgia, rheumatism, skin conditions, indigestion, flatulence, headaches, and gout (8). There are different types of chamomile, among them, Roman chamomile *(Chamaemelum nobile*) and German chamomile *(Matricaria* *recutita*) from the *Asteraceae* family are most popular (9, 10). Roman chamomile has been used for centuries as anti-inflammatory, antioxidant, antibacterial and healing medicine (11). Different types of bioactive compounds are present in chamomile, including phenolic compounds (12, 13). Phenolic compounds, mainly flavonoids, proved to have potency to regulate proliferation and cell death pathways leading to cancer (14) via various mechanisms including cell growth inhibition and apoptosis induction (15). 

To our best knowledge, reports are not available on the antiproliferative activity of *Chamaemelum nobile *(L.) on cancer cells. Our primary screening study indicated that *C. nobile* ethyl acetate fraction had enough potency to inhibit cancer cells growth. Considering these data and knowing that ethyl acetate fraction may contain phenolic compounds with antiproliferative activity, we decided to explore the anticancer effects as well as apopotic mechanism induced by the ethyl acetate fraction obtained from *C. nobile* leaves against three cancer cell lines: MCF-7 (human breast adenocarcinoma), K562 (human erythroleukemia) and SKMEL-3 (human malignant melanoma).

## Experimental

*Chemicals*

Dulbecco’s Modified Eagle’s Medium (DMEM) and fetal bovine serum (FBS) were purchased from Gibco-BRL (Rockville, IN, USA). Anti-Bcl-2 (1:1000), anti-Bax (1:1000), anti- GAPDH and anti-rabbit IgG horseradish peroxidase (HRP) (1:10000) antibodies were purchased from Cell Signaling Technology (Beverly, MA, USA). All other chemicals were from Merck (Darmstadt, Germany) and Sigma-Aldrich (St Louis, MO, USA). ECL advance western blotting detection kit was prepared from General Electric Health Care Life Sciences (Buckinghamshire, UK).

*Plant material and extracts preparation*

*C. nobile* aerial parts were purchased from herbal medicine stores in Tehran, capital of Iran in 2012. It was characterized by herbarium department of Faculty of Pharmacy, Tehran University of Medical Sciences. 20 g of plant powder was extracted sequentially by solvents with different polarities including hexane, chloroform, ethyl acetate and methanol using a maceration method. The process was repeated 3 times with the same plant material but using fresh solvents. After maceration, the extracts were filtered and evaporated to dryness on a rotary evaporator under reduced pressure below 40 ºC. All the extracts were stored at 4 ºC until used for experiments. Yields were 2.66, 2.53, 1.36 and 5.53% for hexane, chloroform, ethyl acetate and methanol fractions, respectively. 

*Cell culture*

MCF-7 (human breast adenocarcinoma), K562 (human erythroleukemia) and SKMEL-3 (human melanoma) cell lines were obtained from the national cell bank of Pasture Institute of Iran (NCBI). Cells were cultured in DMEM with 10% (v/v) FBS, (100 U⁄mL) penicillin and (100 µg ⁄mL) streptomycin under the conditions of 5% CO_2_ at 37 ºC.

*MTT cytotoxicity assay*

The effect of ethyl acetate fraction of *Chamaemelum nobile* on the cytotoxicity of MCF-7, K562 and SK-MEL3 cell lines was determined by MTT assay. The cell proliferation test is based on the ability of the mitochondrial succinate-tertrazolium reductase system to convert yellow tetrazolium salt, MTT *(3-(4,5-dimethylthiazol-2-yl)-2,5 diphenyltetrazolium bromide)* to purple formazan dye. *The cells were added to make 6-8 × 10*^3 ^*cells/well in a 96-well plate including 200 µL of complete culture medium and* incubated for 24 h at 37 °C in 5% CO_2_. Afterwards, cells were exposed to different concentrations of ethyl acetate fraction (0.001- 0.25 mg/mL) and incubated for 24, 48 and 72 h. The solvent DMSO treated cells served as control*. After* *incubation at 37 ºC in a humidified incubator,* cells were treated with MTT *(5 mg/mL)* reagent for 4 h at 37 ºC and then, the medium was removed by aspiration and 200 µL of DMSO was added per well. The absorbance at 545 nm was measured using ELISA Microplate Reader (Star Fax-2100, ST. Louis, USA) .The number of viable cells was proportional to the extent of formazan production. Cell viability was measured as the percentage of absorbance compared with control. The 50% inhibitory concentration (IC_50_) value, the concentration of extract required to inhibit 50% cell growth, was determined from concentration-response curves following a 24, 48 and 72 h exposure times. All experiments were conducted with 3 replicates.

*Flowcytometry analysis*

Cell cycle phase distribution was determined by analytical DNA flowcytometry. MCF-7 cells were incubated for 72 h with 0.001 mg/mL (1/2 IC_50_) of ethyl acetate fraction. Cells were harvested and adjusted to 10^6^ cells/plate in 6-well plates and stained with Propidium Iodide (PI) reagent at 37 ºC for 15 min in the dark. PARTEC flowcytometer (Partec GmbH, Munster, Germany) with Flowjo software was used to analyze DNA content using UV light. The percentage of cells in the various phases was determined, and statistical analysis of data from flowcytometry experiments was carried out.

*Identification of apoptosis by annexin-V/PI staining*

Following treatment, 10^6^ cells were washed in PBS and resuspended in 100 µL of annexin-V-FLUOS labeling solution containing 2 µL annexin-V-FLUOS labeling agent, 2 µL Propidium Iodide (PI) solution and 1 mL incubation buffer to achieve a concentration of 10^6^ cells/mL. Following incubation at 37 ºC for 15 minutes, cells were analyzed by flowcytometry. Annexin-V binds to cells expressing phosphatidyl serine on the outer layer of the cell membrane, and PI stains the cellular DNA of those with a compromised cell membrane. This allows for the discrimination of live cells (unstained with either fluorochrome or PI) from apoptotic (stained with annexin-V) and necrotic cells (stained with PI).

*Western blot analysis*

MCF-7 cells were treated with ethyl acetate fraction at 1/2 IC_50_ concentration for 72 h. Proteins were extracted from distinctively treated cells, collected and lysed in lysis buffer (Tris 62.5 mM (pH 6.8), DTT 50 mM, SDS 10%, glycerol) in the presence of protease inhibitors. Then, equal amounts of protein were heated to 95 ºC, separated in 12% SDS- polyacrylamide gels and transferred to PVDF membranes. The membrane was then blocked for 2 h in TBST (50mmol/L Tris-Cl, pH 7.6, 150 mmol/L NaCl and 0.1% Tween 20) containing 1% (w/v) casein, and then incubated with primary antibodies overnight, followed by incubation with HRP conjugated goat anti-rabbit IgG for 2 h. Blots were then developed using ECL advance western blotting detection kit. The signals from each protein band were normalized against the GAPDH (Glyceraldehyde Phosphate Dehydrogenase) content using the polyclonal anti-GAPDH antibody. The expression level of control was designated value “1”, and thereby the expression ratios of the treatments were expressed in relation to the control.

Statistical analysis 

*IC*_50_ *values* were calculated by non-linear regression analysis with Graph Pad Prism 6.0. Results were expressed as the mean ± SE of at least triplicate determinations, and statistical comparisons were based on ANOVA followed by the Tukey’s post test. *P *< 0.05 was considered to be significant.

## Results and Discussion

**Figure 1:**
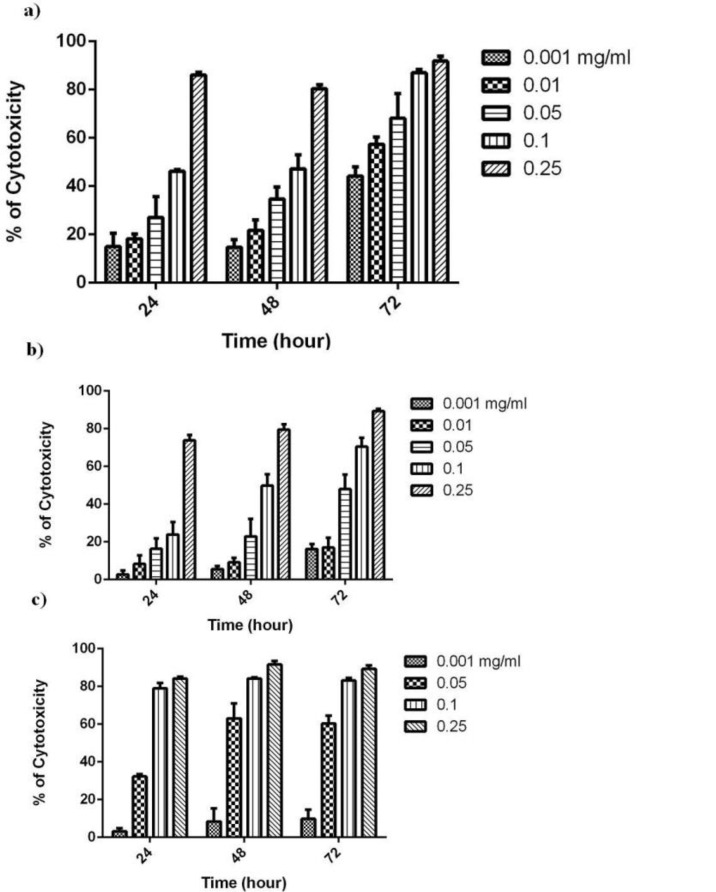
Cytotoxic effects of ethyl acetate extract on a) MCF-7, b) SK-MEL-3 and c) K562 cells after 24, 48 and 72 h treatment. Cells were treated with different concentrations of extract (0.001-0.25 mg/mL) .Values are presented as mean ± SE of three independent experiments

It has been shown that German chamomile extracts caused minimal growth inhibitory responses in normal cells, whereas a significant decrease in cell viability was observed in various human cancer cell lines. In this regard, German chamomile contains polyphenols such as apigenin possessing high anticancer activity (16). Furthermore, chamomile exposure resulted in differential apoptosis in cancer cells but not in normal cells at similar concentrations (17). We have recently evaluated the antiproliferative activity of different fractions obtained from *C. nobile* (Roman chamomile) in human oral cancer cells (BHY) (data not shown). Our results indicated that chloroform as well as ethyl acetate fractions both had considerable and similar IC_50_ values, particularly after 72 h incubation (0.05 *vs*. 0.09 mg/mL). Additionally, previous studies provide evidence that polar chemicals including phenolic compounds found in chamomile species can exert health beneficial effects including tumor-suppressive property (16, 18). Considering this supposition and that the ethyl acetate fraction contains polar bioactive compounds, we decided to evaluate the antiproliferative effects of ethyl acetate fraction obtained from aerial parts of *C. nobile*. For this purpose, we examined the effect of different concentrations of ethyl acetate fraction on MCF-7, K562 and SK-MEL3 cells cytotoxicity using the MTT assay at 24, 48 and 72 h. Ethyl acetate extract exhibited remarkable growth inhibitory activity as illustrated by the concentration-dependent curves in Figure 1 at different times. The IC_50 _results are summarized in Table 1. Considering all IC_50_ values, the best cytotoxic effect obtained after 72 h treatment of MCF-7 cells. Additionally, the IC_50_ value for the ethyl acetate fraction obtained after 72 h treatment of human gingival fibroblast cells (HGF) as normal cell line (IC_50_ = 0.05 mg/mL). As the results shown, the IC_50_ value in normal cell line was higher than that of cancer cell lines showing selectivity between cancer and non-cancer cells. In this regard, the selectivity index in MCF-7 is much higher than in SKMEL-3 or K562 cells. Our results are in close agreement with Guimaraes *et al*. who studied the cytotoxic effect of decoction, infusion and extract of *C. nobile* on the growth of human tumour cells (19). From these results, it is worth mentioning that the anticancer compounds extracted from *C. nobile* are concentrated in ethyl acetate fraction.

**Table 1:** IC_50_ values (mg of extract/mL) for antiproliferative activity of *Chamaemelum nobile* ethyl acetate extract towards MCF-7, K562 and SKMEL3 cells a.

**Cells**	**Time**	**Ethyl acetate fraction**
	24 h	0.09(0.07-0.1)
MCF-7	48h	0.09(0.07-0.1)
	72h	0.002(0.002-0.004)
	24 h	0.06(0.05-0.07)
K562	48h	0.04(0.03-0.04)
	72h	0.04(0.03-0.04)
	24 h	0.15 (0.14-0.17)
SKMEL-3	48h	0.1(0.09-0.11)
	72h	0.04(0.04-0.05)

a **MCF-7**: Human breast adenocarcinoma, **K562** : Human erythroleukemia,** SK-MEL-3**: Human melanoma cell lines. Data are expressed as mean of three separate experiments run in triplicate and 95% confidence intervals**.**

**Figure 2:**
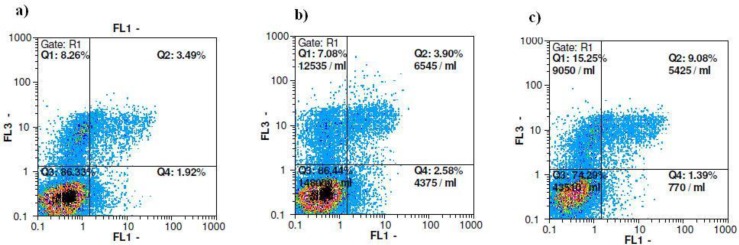
Flowcytometric analysis of annexin-V/PI to quantify extract-induced apoptosis in MCF-7 cells. a) Dot plot of MCF-7 cells as control. b) Dot plot of MCF-7 cells treated with DMSO for 72 h. c) Dot plot of MCF-7 cells treated with 1/2 IC_50_ concentration of ethyl acetate fraction for 72 h

Next, we confirmed the antiproliferative effect of ethyl acetate fraction on breast cancer cells. As shown in Table 2, exposure to 0.001 mg/mL of ethyl acetate fraction slightly inhibited cell division of PI- stained MCF-7 cells after 72 h. Cell cycle analysis of MCF-7 cells-treated with ½ IC_50_ concentration of the ethyl acetate fraction, exhibited an inconsiderable increase in sub-G1 population from 4.27% to 5.8% after 72 h treatment. Concomitantly, the percentage of cells in the G2 phase also increased marginally from 28.6 ± 2.5% in control cells to 32.7±4.4% in the ½ IC_50_ treated group suggesting a slight G2/M phase arrest (Table 2).In order to explore whether ethyl acetate fraction showed cytotoxicity to MCF-7 cells through inducing apoptosis, the cells were stained with annexin-V/PI and analyzed by flowcytometry. In this assay, detection of the externalization of phosphatidylserine was performed. Based on Table 3, there is a pattern of cell population shifting from viable to late apoptosis/necrosis in MCF-7 cells. The percentage of late apoptotic cells in MCF-7 increased gradually from 3.7% in solvent control group up until 8.6% in ½ IC_50_ of the treatment group. A similar pattern can be seen in the necrotic cells as well, according to Figure 2. The results obtained from annexin-V/PI assay indicated that ethyl acetate fraction showed antitumor activities through inducing mainly late apoptotic and necrotic cell death.

**Table 2:** Effect of ethyl acetate fraction on cell cycle progression with respect to control^a^.

		** MCF-7**		
** Treatment**	**Sub-G1** **(mean SE** **(**	**G**_0_ **/G**_1_ **(mean ± SE)**	**S** **(mean ± SE)**	**G**_2_ **/M** **(mean ± SE)**
**Control**	4.27 ± 1.2	31.3 ± 3.3	23.9 ± 0.75	28.56 ± 2.5
**Ethyl acetate fraction (1/2 IC**_50_ **)**	5.8 ± 1.9	31.39 ± 1.4	22.53 ± 0.28	32.7 ± 4.4

a At the indicated cell, distribution of the cells in sub-G1, G_o_/G1, S and G2/M phase was analyzed by flowcytometry. Results are expressed as total cells. Data represent means of triplicate experiment.

**Table 3:** Percentage of breast adenocarcinoma cells in each state after treatment with ethyl acetate fraction at 0.001 mg/mL for 72 h of incubation

**Compound**	** Vital cells (%) ** ** An–/PI–**	** Early apoptosis (%) ** ** An+/PI–**	**Late apoptosis (%) An+/PI+**	** Necrosis (%) An– ** ** /PI+**
**Ethyl acetate fraction (1/2 IC**_50_ **)**	73.5 ± 4.9	1.4 ± 0.2	8.6 ± 2.7	14.3 ± 0.92
**Control**	83.5 ± 1.6	2.2 ± 0.3	3.7 ± 0.1	6.2 ± 0.84

**Figure 3:**
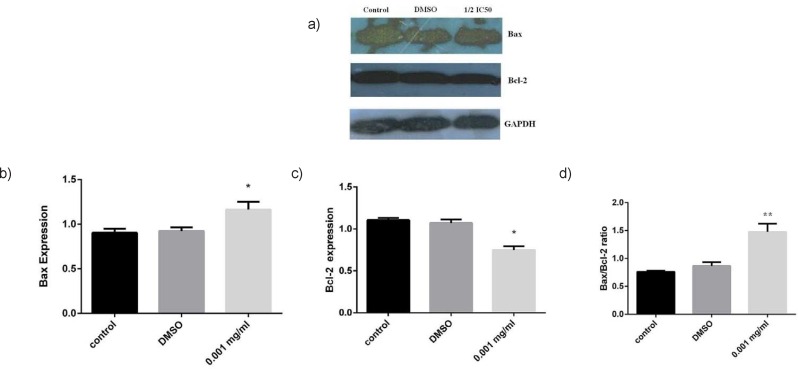
Expression analysis of apoptosis associated proteins by Western blot analysis. MCF-7 cells were treated by ethyl acetate fraction at ½ IC50 concentration a) Immunoblot showing the expression levels of Bax, Bcl-2 and GADPH. Statistical analysis of b) Bax, c) Bcl-2 expression and d) Ratios of Bax/Bcl-2 compared with control after 72 h treatment. The results of three independent experiments are presented as mean ± standard error. * *p*<0.05 and ** *p*<0.01 indicate significant differences of each group with the control group

 It has been reported that anticancer effects of major phenolic compounds extracted from *Matricaria chamo*mile (German chamomile) in different cancer cells were based on the activation of the classical apoptosis response, particularly the mitochondrial pathway of the apoptosis (16, 20). These studies prompted us to elucidate the mechanism of cytotoxic effects of ethyl acetate fraction. To evaluate the potential mediators of ethyl acetate fraction-induced cell damage, we analyzed Bax/Bcl-2 proteins ratio as cell apoptosis markers. The cells were exposed to 0.001 mg/mL of the extract (1/2 IC_50_ concentration) for 72 h. As indicated in Figure 3, Bax protein was remarkably increased in ethyl acetate-treated cells, while the Bcl-2 protein significantly (𝑃 < 0.05) decreased. However, it is important to mention that the Bcl-2 is highly expressed in MCF-7 cells. Consequently, there was a statistically significant increase (𝑃 < 0.05) in the Bax / Bcl-2 protein ratio in the cells exposed to 0.001 mg/mL of the extract (Figure 3). This increase is important because it has been recently proposed that the ratio of Bax to Bcl-2 may govern the sensitivity of cells to apoptotic stimuli from anticancer agents (21). These data also supported our annexin –V and cell cycle analysis results suggesting apoptosis induction by treatment of *C. nobile* ethyl acetate fraction in MCF-7 cells through the blockade of cell cycle progression. These results are in line with data reported by Srivastava *et al*. who demonstrated a notable apoptotic effect of methanolic extract in cancer cell lines which was due to flavonid compounds, specially apigenin derivatives (16).

This study proposes *C. nobile* ethyl acetate fraction as a promising anti-cancer agent especially in treating breast cancer. Ethyl acetate fraction induced apoptosis in MCF-7 cells by activating the mitochondrial death pathway via the involvement of G2/M phase arrest. However, we do not know whether apoptosis induced by ethyl acetate fraction relies on a single or combined effects of different compounds detected in this extract. Finally, *C. nobile* ethyl acetate extract deserves further investigation to fully explain the mechanism of apoptosis inducing effect and the chemical constituents involved in this activity.
